# Diffusion weighted imaging of the breast: Performance of standardized breast tumor tissue selection methods in clinical decision making

**DOI:** 10.1371/journal.pone.0245930

**Published:** 2021-01-25

**Authors:** M. Wielema, P. E. Sijens, H. Dijkstra, G. H. De Bock, I. G. van Bruggen, J. E. Siegersma, E. Langius, R. M. Pijnappel, M. D. Dorrius, M. Oudkerk

**Affiliations:** 1 Department of Radiology, University Medical Center Groningen, University of Groningen, Groningen, the Netherlands; 2 Department of Epidemiology, University Medical Center Groningen, University of Groningen, Groningen, the Netherlands; 3 Department of Radiotherapy, University Medical Center Groningen, University of Groningen, Groningen, the Netherlands; 4 Department of Radiology, Isala Hospital, Zwolle, the Netherlands; 5 Department of Radiology, University Medical Center Utrecht, Utrecht University, Utrecht, the Netherlands; 6 Faculty of Medical Sciences, University of Groningen, Groningen, the Netherlands; 7 Institute of Diagnostic Accuracy, Groningen, the Netherlands; Medical University of Vienna, AUSTRIA

## Abstract

**Objectives:**

In breast diffusion weighted imaging (DWI) protocol standardization, it is recently shown that no breast tumor tissue selection (BTTS) method outperformed the others. The purpose of this study is to analyze the feasibility of three fixed-size breast tumor tissue selection (BTTS) methods based on the reproducibility, accuracy and time-measurement in comparison to the largest oval and manual delineation in breast diffusion weighted imaging data.

**Methods:**

This study is performed with a consecutive dataset of 116 breast lesions (98 malignant) of at least 1.0 cm, scanned in accordance with the EUSOBI breast DWI working group recommendations. Reproducibility of the maximum size manual (BTTS1) and of the maximal size round/oval (BTTS2) methods were compared with three smaller fixed-size circular BTTS methods in the middle of each lesion (BTTS3, 0.12 cm^3^ volume) and at lowest apparent diffusion coefficient (ADC) (BTTS4, 0.12 cm^3^; BTTS5, 0.24 cm^3^). Mean ADC values, intraclass-correlation-coefficients (ICCs), area under the curve (AUC) and measurement times (sec) of the 5 BTTS methods were assessed by two observers.

**Results:**

Excellent inter- and intra-observer agreement was found for any BTTS (with ICC 0.88–0.92 and 0.92–0.94, respectively). Significant difference in ADCmean between any pair of BTTS methods was shown (p = <0.001–0.009), except for BTTS2 vs. BTTS3 for observer 1 (p = 0.10). AUCs were comparable between BTTS methods, with highest AUC for BTTS2 (0.89–0.91) and lowest for BTTS4 (0.76–0.85). However, as an indicator of clinical feasibility, BTTS2-3 showed shortest measurement times (10–15 sec) compared to BTTS1, 4–5 (19–39 sec).

**Conclusion:**

The performance of fixed-size BTTS methods, as a potential tool for clinical decision making, shows equal AUC but shorter ADC measurement time compared to manual or oval whole lesion measurements. The advantage of a fixed size BTTS method is the excellent reproducibility. A central fixed breast tumor tissue volume of 0.12 cm^3^ is the most feasible method for use in clinical practice.

## Introduction

Breast Dynamic Contrast Enhanced MRI (DCE-MRI) has the highest negative predictive value of all imaging diagnostic techniques in the exclusion of breast malignancy [1, 2]. However, overlap in enhancement patterns of malignant and benign breast lesions exists. Diffusion Weighted Imaging (DWI) in addition to DCE-MRI improves the specificity of breast MRI and can prevent unnecessary biopsies in benign lesions [3, 4]. However, DWI cannot be used as a stand-alone parameter [5]. DWI measures the diffusion of hydrogen protons in a voxel due to Brownian motion and is most often quantified in a mono-exponential model, using the apparent diffusion coefficient (ADC). There are initiatives to improve and standardize DWI protocols, however, further research is needed [6–8]. In image analysis, literature is inconclusive on the influence of breast tumor tissue selection (BTTS) methods on the accuracy of DWI in the discrimination of benign from malignant lesions. Some authors state that the applied tumor tissue selection method (by definition of a region of interest) influences the ADC outcome [9–12], which thereby could affect the differentiation between malignant and benign breast lesions [13]. However, no superior BTTS method was found, due to the high heterogeneity in the available data, in a recent meta-analysis [14]. Therefore, there is a need to compare the accuracy of the five most used BTTS methods in the same data set, acquired with a robust MRI protocol. Furthermore, data is lacking on which method is most feasible to implement as measured by the amount of time needed to perform the assessment.

The purpose of this study is to evaluate the reproducibility, time measurement and accuracy of fixed size and shape breast tumor tissue selection methods compared to conventionally drawn tumor tissue delineation methods. For the radiologist, a standard fixed size BTTS method would be expected to save time and improve robustness of breast lesion ADC measurement.

## Materials and methods

### Patient population

A consecutive sample of 105 women (mean 48 years (range: 23–75)) with 116 enhancing breast masses (98 malignant) were included between April 2010 and June 2015. "The medical ethical committee of the University Medical Center Groningen approved the study and waived the need for informed consent due to the retrospective nature of the study (METc Nr: 2016/379). However, all participants were checked for registration in the local legal “opt-out of research system”. None of the included participants opted out". Lesion diameter was at least 1.0 cm, with an area of ≥ 0.8 cm^2^. Indications for breast MRI consisted of pre-operative/pre-chemotherapy evaluation, problem solving and screening of high-risk women. Non-mass enhancement lesions were excluded to reduce partial volume effect based on the known limited value of DWI in non-mass lesions [15]. Exclusion criteria were: previous breast malignancy, breast implants and simple cysts. Final diagnosis was acquired by pathology or follow-up of at least 2 years.

### Data acquisition

MRI examinations were performed using a 1.5 Tesla (T) system (Magnetom Avanto, Siemens Healthineers), with a circularly polarized bilateral breast coil (Siemens Healthineers), with patients in prone position. The MRI protocol consisted of pre-contrast T1, T2, DWI and 5–7 DCE-T1-weighted series. DWI was performed with single shot—echo planar imaging (SS-EPI) with spectral attenuated inversion recovery (SPAIR) fat suppression and b-values of 0, 50, 200, 500, 800 and 1000 s/mm^2^ (TR/TE 9300/91 ms, FOV 170 x 340 mm^2^, matrix 192 x 384, bandwidth 1628 Hz/pixel, slice thickness 4 mm, inter-slice gap 2 mm). Acquisition time of DWI was 5 minutes and 15 seconds. In this study, the automatically calculated ADC maps with b = 0 and b = 1000 s/mm^2^ were used because of their proven high accuracy in literature [8, 13].

### Image analysis

**Fig 1:**
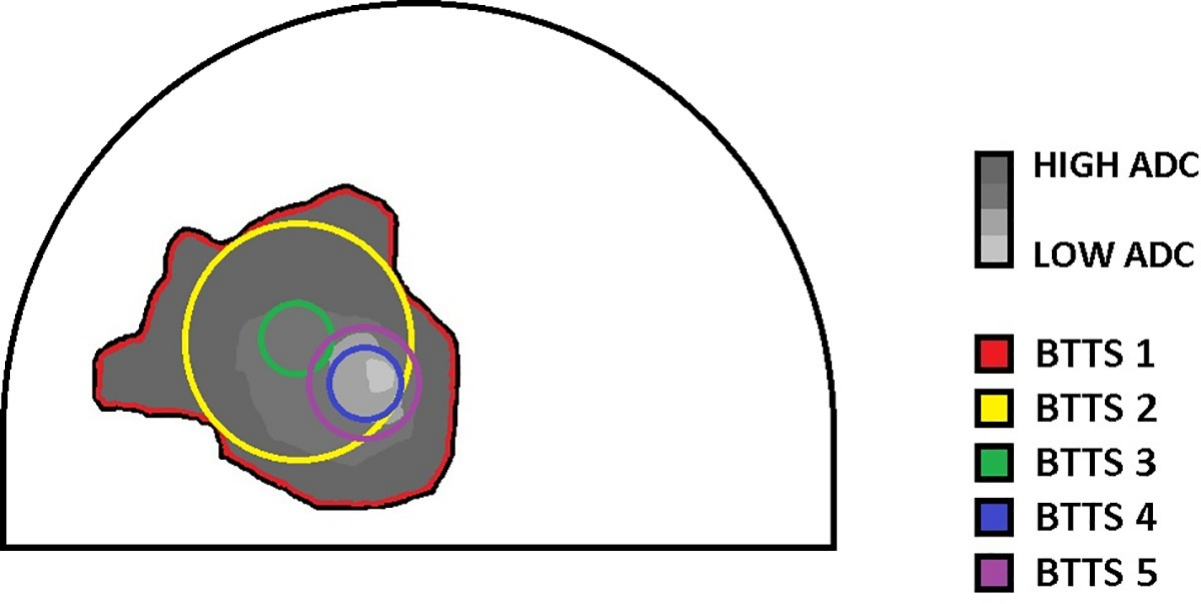
Schematic overview of the five breast tumor tissue selection methods in a breast lesion on diffusion weighted imaging. BTTS = Breast tumor tissue selection, ADC = Apparent diffusion coefficient.

A radiologist with 8 years of experience in breast MRI (MDD) and a radiologist in training (MW) localized the slice with the highest lesion diameter on DCE-T1 images in consensus. Two observers (observer 1 (IVB), technical medicine physicist in training, observer 2 (JES) clinical physicist in training) independently positioned the 5 BTTSs in each lesion. Both observers were trained and tested in tumor delineation in an independent sample of 25 breast MRI tumor supervised cases. Observers were blinded to all clinical data. Observer 1 repeated all measurements after one month. Fig 1 shows the BTTS methods that were compared: BTTS1: Manual, whole breast tumor tissue selection volume; BTTS2: Oval shaped, whole breast tumor tissue selection, encompassing as much of the lesion as possible while staying within its borders; BTTS3: Standardized fixed circle of 0.3 cm^2^ (x 4 mm slice thickness = volume of 0.12 cm^3^) in the middle of the lesion; BTTS4: standard circular fixed area of 0.3 cm^2^ (volume of 0.12 cm^3^) and BTTS5: standard circular fixed area of 0.6 cm^2^ (volume of 0.24 cm^3^). Both BTTS4 and BTTS5 were positioned to obtain the lowest mean ADC, as an indicator of the most cellular part of the lesion, while avoiding necrotic parts. BTTS1 was positioned on the DCE-T1 series and copied to the ADC-map. BTTS2-5 were positioned on the ADC map. In several cases, DWI series and DCE-T1 series were not linked correctly. To correct for this registration mismatch, BTTS1 was manually moved up or down in the same slice, to where the lesion was clearly seen. Dedicated software was used for image analysis: Multiview (Hologic).

### Time measurements

Measurement times were registered using an online stopwatch tool (http://stopwatch.online-timers.com/online-stopwatch). Time measurement of the BTTS methods only consisted of BTTS placement. Slice selection was not included in the measurement, since it is similar for all methods. As planned on forehand, the first 10 consecutive cases were used to train the observers in using the online stopwatch tool and were not included in the time measurement analysis. The next 50 consecutive cases were timed for both observers separately and included in the data analysis. Time measurements were performed in the first session of the two tumor tissue delineation sessions.

### Statistical analysis

For statistical analysis IBM SPSS Statistics 23 and MedCalc (version 12.5.0.0) were used. Average ADC (ADCmean) and minimal ADC (ADCmin) values of BTTS1-5 were measured for each breast lesion. The size (area, mm^2^) of BTTS1-2 was recorded. Average and minimal ADC’s of benign and malignant lesion groups were separately tested for normal distribution using Shapiro-Wilk test. Due to the non-normal distribution, median and interquartile ranges (IQR) were used in further statistical testing. ADC values of benign and malignant lesions were compared for each BTTS method using Mann-Whitney U tests (for unrelated samples). Wilcoxon signed rank test (for related samples) was used to compare ADC values between BTTS methods. Intra- and inter-observer agreement was calculated by using the Intraclass Correlation Coefficient (ICC) of measured ADC values for each BTTS method. In the discrimination between benign and malignant lesions of the different BTTS methods, the area under the ROC curve (AUC) ±standard error (SE) of ADC was measured for each BTTS method per observer. The method of DeLong et al. was used to compare the AUC’s (using the AUC’s ±SE) [16]. Time measurements were normally distributed and compared using repeated measurement ANOVA for both observers separately. Further post-hoc pairwise comparison was performed with a Bonferroni post hoc test. A p-value of <0.05 was considered to indicate a statistically significant difference.

## Results

### Lesions characteristics

**Fig 2:**
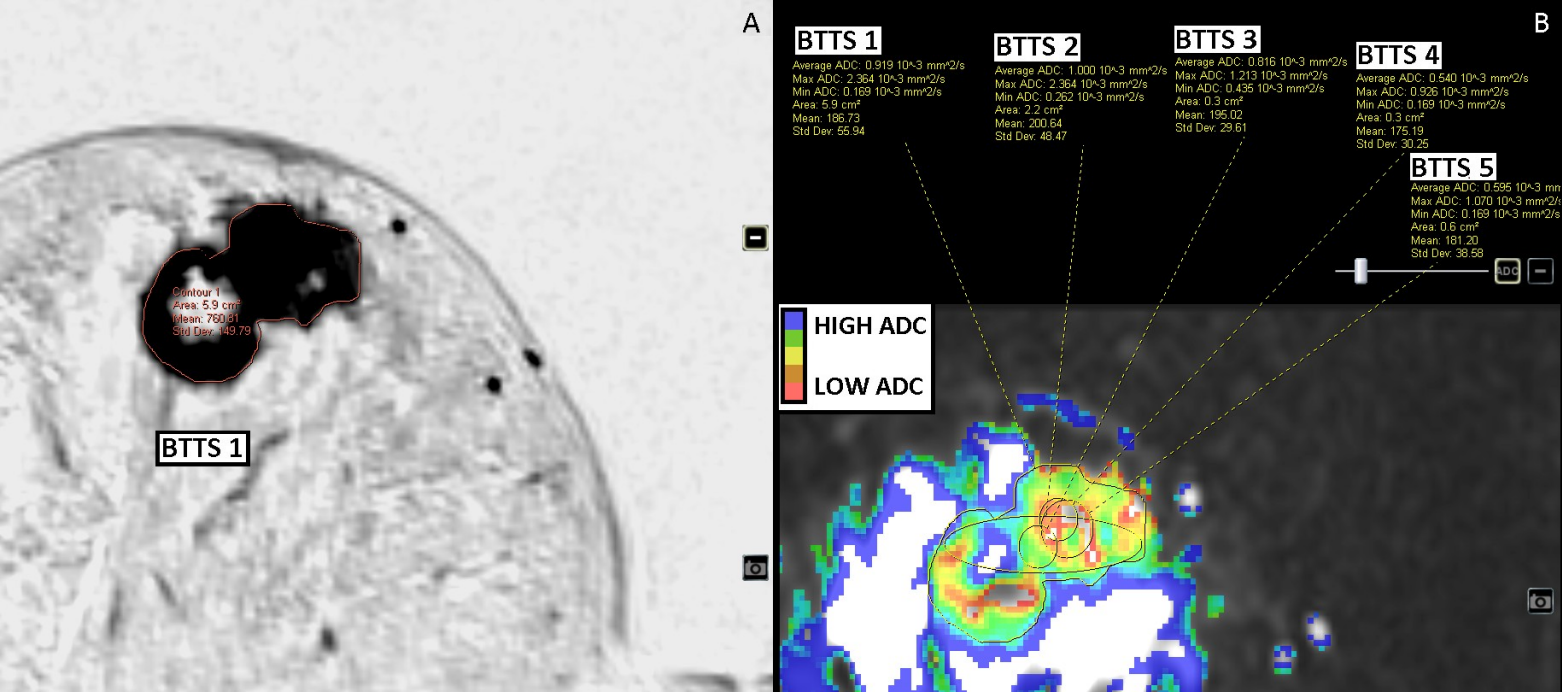
Invasive ductal carcinoma of the right breast, ER and PR positive, HER2-neu negative. DCE-T1 subtracted images (inverted) (A) and DWI b0-1000 images (B).

**Fig 3:**
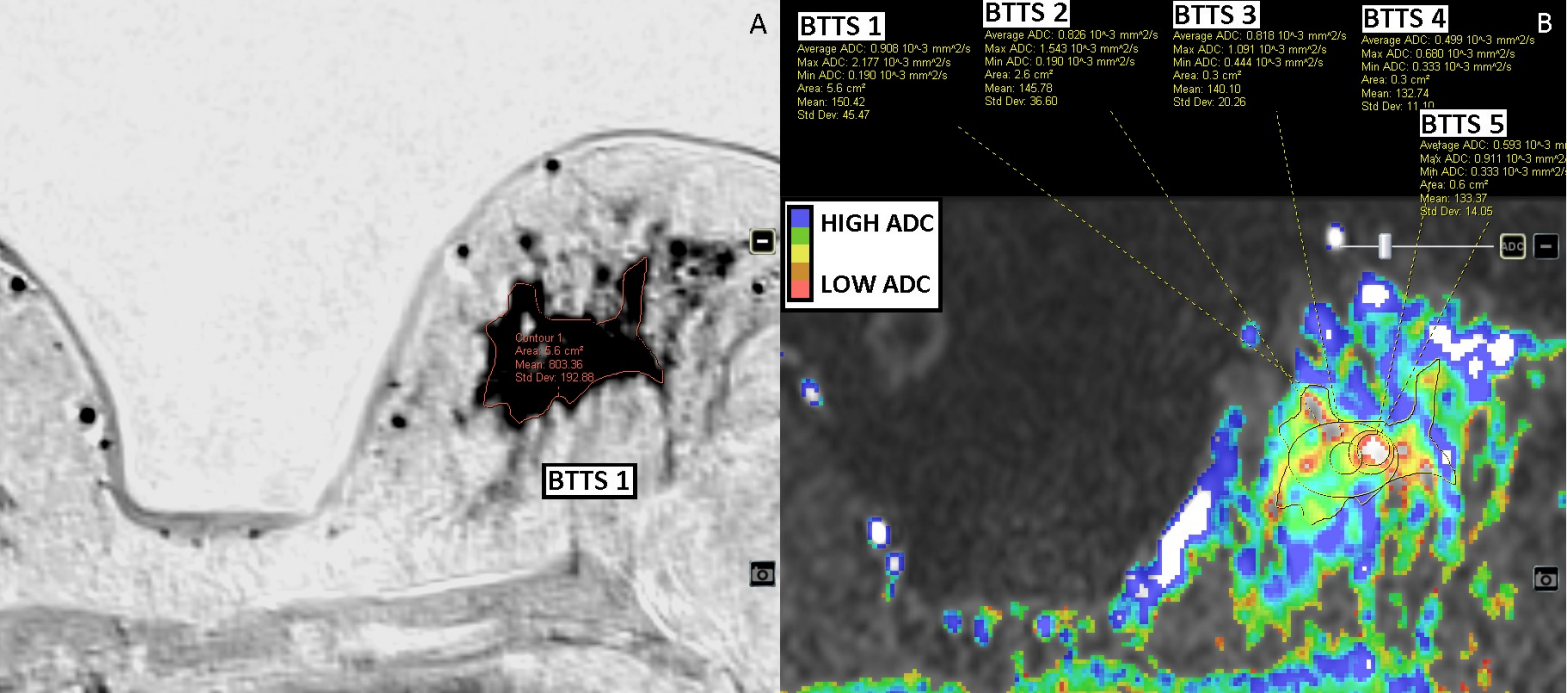
Invasive lobular carcinoma of the left breast, ER positive, PR negative and HER2-neu positive. Surrounding lobular carcinoma in situ. DCE-T1 subtracted images (inverted) (A) and DWI b0-1000 images (B).

**Fig 4:**
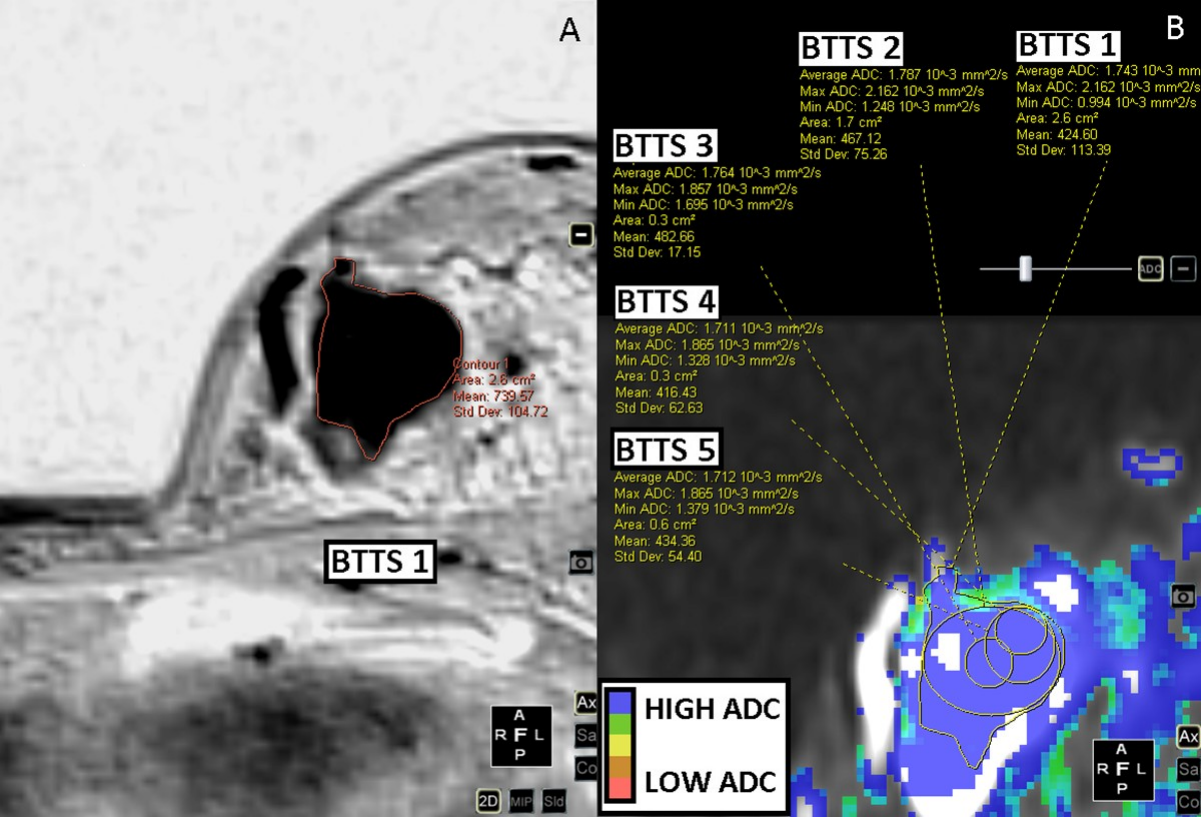
Benign phyllodes tumor of the left breast. DCE-T1 subtracted images (inverted) (A) and DWI b0-1000 images (B).

**Fig 5:**
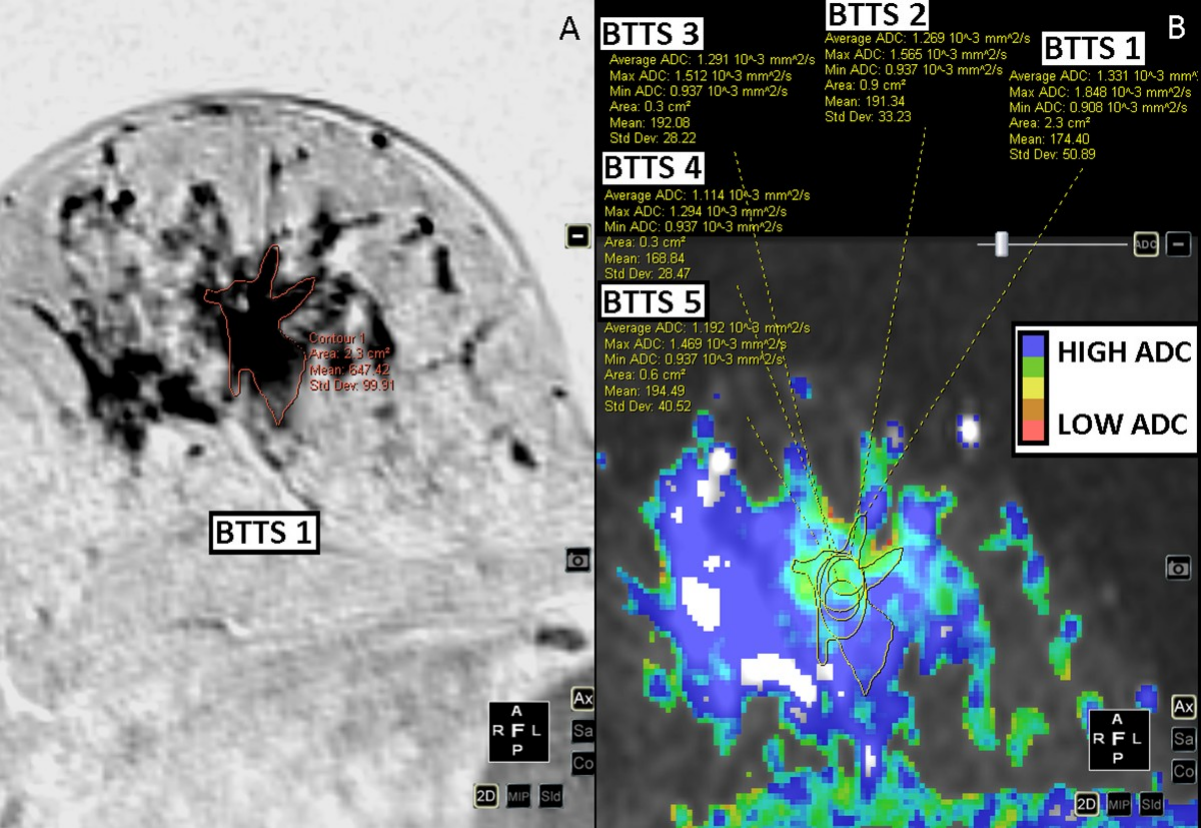
Sclerosing adenosis, columnar cell changes and apocrine hyperplasia of the right breast. MRI guided biopsy proven and unchanged in 4 years of follow-up. DCE-T1 subtracted images (inverted) (A) and DWI b0-1000 images (B). BTTS = Breast tumor tissue selection.

Out of the 116 enhancing breast lesions 98 were malignant and 18 benign. Malignant lesions consisted of: invasive carcinoma no special type (n = 80); invasive lobular carcinoma (n = 13); ductal carcinoma in situ (n = 1); invasive papillary carcinoma (n = 1); malignant phyllodes tumor (n = 1); mucinous carcinoma (n = 1) and medullary carcinoma (n = 1). Benign lesions were: fibroadenoma (n = 8); sclerosing adenosis/columnar cell changes/apocrine metaplasia (n = 6); chronic inflammation (n = 3); and benign phyllodes (n = 1). For BTTS1, the mean area was 4.1 ± 3.9 cm^2^ for malignant lesions with a range between 0.8 cm^2^ and 23.4 cm^2^; for benign lesions the mean area was 3.7 ± 4.2 cm^2^ (range: 0.8–17.8 cm^2^). For BTTS2 the mean area was 2.3 ± 2.4 cm^2^ (range: 0.6–19.1 cm^2^) and 1.8 ± 2.1 cm^2^ (range: 0.6–12.2 cm^2^) for malignant and benign lesions, respectively. BTTS 3–5 were of standard size and shape. Figs 2–5 show examples of the BTTS methods in both malignant and benign breast lesions.

### ADC values

Table 1 shows the mean lesion ADC (ADCmean, mm^2^/s) and, for a comparison, also the minimum pixel ADC values (ADCmin, mm^2^/s) for the five BTTS methods. Values are given as median (± IQR) ADC, due to the non-normal distribution of the data. Both ADCmean (p<0.00) and ADCmin (p<0.00–0.038) showed significantly different values for benign vs. malignant lesions for each BTTS method. Median values of ADCmean for benign lesions ranged 1.29–1.74 mm^2^/s and 0.72–1.11 mm^2^/s for malignant lesions. Statistical analysis showed a significant difference in ADCmean between any pair of the 5 BTTS methods (p = 0.000–0.009), except for BTTS2 vs. BTTS3 for observer 1 (p = 0.10) (Table 2).

**Table 1:** Median (± IQR) ADC values (mm^2^/s) for each BTTS method, average (ADCmean) and minimal (ADCmin) values. Results are presented per observer (and session) and for malignant and benign lesions separately. P-values indicate a significant difference between malignant and benign lesions for each BTTS method, per observer.

	Malignant: Median ADC (± IQR) (mm2/s) Observer 1 1^st^ read	Benign: Median ADC (± IQR) (mm2/s) Observer 1 1^st^ read	p-value	Malignant: Median ADC (± IQR) (mm2/s) Observer 1 2^nd^ read	Benign: Median ADC (± IQR) (mm2/s) Observer 1 2^nd^ read	p-value	Malignant: Median ADC (± IQR) (mm2/s) Observer 2	Benign: Median ADC (± IQR) (mm2/s) Observer 2	p-value
**BTTS 1 ADCmean**	1.11 (±0.31)	1.57 (±0.54)	<0.001	1.10 (±0.31)	1.63 (±0.64)	<0.001	1.13 (±0.24)	1.65 (±0.56)	<0.001
**BTTS 2 ADCmean**	1.06 (±0.29)	1.60 (±0.54)	<0.001	1.03 (±0.31)	1.63 (±0.55)	<0.001	1.06 (±0.27)	1.69 (±0.64)	<0.001
**BTTS 3 ADCmean**	1.02 (±0.42)	1.59 (±0.51)	<0.001	0.95 (±0.36)	1.69 (±0.58)	<0.001	0.97 (±0.36)	1.74 (±0.66)	<0.001
**BTTS 4 ADCmean**	0.72 (±0.32)	1.33 (±0.47)	<0.001	0.75 (±0.30)	1.39 (±0.44)	<0.001	0.74 (±0.26)	1.29 (±0.75)	<0.001
**BTTS 5 ADCmean**	0.81 (±0.30)	1.41 (±0.44)	<0.001	0.82 (±0.30)	1.52 (±0.51)	<0.001	0.83 (±0.29)	1.55 (±0.69)	<0.001
**BTTS 1 ADCmin**	0.21 (±0.43)	0.43 (±0.59)	0.028	0.20 (±0.40)	0.42 (±0.49)	0.012	0.25 (±0.47)	0.52 (±0.67)	0.038
**BTTS 2 ADCmin**	0.42 (±0.45)	0.77 (±0.53)	<0.001	0.37 (±0.44)	0.64 (±0.62)	<0.001	0.45 (±0.41)	0.72 (±0.58)	0.001
**BTTS 3 ADCmin**	0.71 (±0.31)	1.29 (±0.59)	<0.001	0.70 (±0.30)	1.33 (±0.41)	<0.001	0.72 (±0.32)	1.40 (±0.65)	<0.001
**BTTS 4 ADCmin**	0.39 (±0.46)	0.72 (±0.69)	0.001	0.39 (±0.46)	0.60 (±0.58)	0.015	0.46 (±0.46)	0.76 (±0.81)	0.005
**BTTS 5 ADCmin**	0.42 (±0.44)	0.81 (±0.69)	<0.001	0.40 (±0.48)	0.69 (±0.68)	0.002	0.49 (±0.44)	0.74 (±0.87)	0.006
**Size of BTTS 1**	2.70 (±3.3)	1.65 (±2.6)		2.60 (±2.9)	1.40 (±2.4)		2.70 (±2.4)	1.65 (±2.5)	
**Size of BTTS 2**	1.55 (±1.7)	0.95 (±1.1)		1.60 (±1.5)	0.90 (±1.3)		1.60 (±1.4)	1.10 (±1.2)	

BTTS = breast tumor tissue selection, ADC = apparent diffusion coefficient (mm^2^/s), IQR = interquartile range.

**Table 2:** Comparison of ADCmean (mm^2^/s) between BTTS methods using Wilcoxon signed rank test, a non-parametric test for related samples.

	Observer 1: p-value	Observer 2: p-value
**BTTS1 vs. BTTS 2**	0.001	<0.001
**BTTS1 vs. BTTS3**	0.009	<0.001
**BTTS1 vs. BTTS4**	<0.001	<0.001
**BTTS1 vs. BTTS5**	<0.001	<0.001
**BTTS2 vs. BTTS3**	0.101*	0.001
**BTTS2 vs. BTTS4**	<0.001	<0.001
**BTTS2 vs. BTTS5**	<0.001	<0.001
**BTTS3 vs. BTTS4**	<0.001	<0.001
**BTTS3 vs. BTTS5**	<0.001	<0.001
**BTTS4 vs. BTTS5**	<0.001	<0.001

*No significant difference: p >0.05. BTTS = breast tumor tissue selection.

### Inter- and intra-observer variability

Table 3 shows the inter- and intra- observer agreement in lesion ADC values obtained in the 118 breast lesions, per BTTS method. Excellent inter- and intra-observer agreement was found for BTTS2 and BTTS5 (ICC >0.9). Good agreement was found for the other BTTS methods. ADCmean showed higher inter-observer and intra-observer agreement than ADCmin.

**Table 3:** Inter-observer and intra-observer agreement, shown intraclass correlation coefficient (ICC) and confidence interval (CI) for the mean and minimal ADC measurements of the BTTS methods.

	Inter-observer ICC (CI)	Intra-observer ICC (CI)
**BTTS 1 ADCmean**	0.899 (0.852–0.930)	0.931 (0.901–0.952)
**BTTS 2 ADCmean**	0.906 (0.864–0.935)	0.940 (0.914–0.959)
**BTTS 3 ADCmean**^**a**^	0.882 (0.829–0.918)	0.922 (0.887–0.946)
**BTTS 4 ADCmean**^**a**^	0.882 (0.830–0.919)	0.939 (0.912–0.958)
**BTTS 5 ADCmean**^**a**^	0.917 (0.880–0.942)	0.924 (0.890–0.947)
**BTTS 1 ADCmin**	0.767 (0.664–0.838)	0.845 (0.776–0.892)
**BTTS 2 ADCmin**	0.769 (0.667–0.840)	0.864 (0.804–0.906)
**BTTS 3 ADCmin**^**a**^	0.875 (0.820–0.914)	0.911 (0.871–0.939)
**BTTS 4 ADCmin**^**a**^	0.764 (0.660–0.836)	0.796 (0.706–0.859)
**BTTS 5 ADCmin**^**a**^	0.742 (0.629–0.821)	0.823 (0.744–0.877)

BTTS = breast tumor tissue selection, ICC = intraclass correlation coefficient, CI = confidence interval, ^a^ = fixed size.

### Accuracy analysis

In the analysis of the influence of the BTTS methods on the accuracy, as measured by the area under the ROC curve (AUC), ADCmean showed higher AUCs compared to ADCmin (Table 4). BTTS methods measuring ADCmean showed comparable AUCs with the highest AUC of 0.889–0.911 for BTTS2 (Tables 4 and 5). Only for observer 1 BTTS1 and BTTS2 showed significantly different AUCs in the second reading session, due to a lower AUC for BTTS1 compared to the first reading session and compared to observer 2.

**Table 4:** Area under the ROC curve for all observers per BTTS method. Results for ADCmean and ADCmin separately.

n = 116	AUC (±SE) Observer 1 (1^st^ read)	AUC (±SE) Observer 1 (2^nd^ read)	AUC (±SE) Observer 2
**BTTS 1 ADCmean**	0.881 (±0.046)	0.858 (±0.051)	0.868 (±0.058)
**BTTS 2 ADCmean**	0.911 (±0.032)	0.910 (±0.032)	0.889 (±0.050)
**BTTS 3 ADCmean**^**a**^	0.862 (±0.051)	0.858 (±0.061)	0.881 (±0.047)
**BTTS 4 ADCmean**^**a**^	0.852 (±0.071)	0.830 (±0.081)	0.763 (±0.090)
**BTTS 5 ADCmean**^**a**^	0.856 (±0.066)	0.853 (±0.069)	0.842 (±0.069)
**BTTS 1 ADCmin**	0.664 (±0.074)	0.688 (±0.076)	0.654 (±0.078)
**BTTS 2 ADCmin**	0.772 (±0.066)	0.775 (±0.067)	0.749 (±0.066)
**BTTS 3 ADCmin**^**a**^	0.807 (±0.068)	0.842 (±0.067)	0.842 (±0.060)
**BTTS 4 ADCmin**^**a**^	0.755 (±0.072)	0.681 (±0.079)	0.707 (±0.082)
**BTTS 5 ADCmin**^**a**^	0.779 (±0.072)	0.732 (±0.083)	0.704 (±0.083)

BTTS = breast tumor tissue selection, ADC = apparent diffusion coefficient (mm^2^/s), AUC = area under the ROC curve, ROC = receiver operating characteristic, SE = Standard Error

^a^ = fixed size.

**Table 5:** Comparison of AUC’s for all observers per BTTS method. Results are presented for ADCmean.

	Observer 1 (1^st^ read): p-value	Observer 1 (2^nd^ read): p-value	Observer 2: p-value
**BTTS1 vs. BTTS 2**	0.162	0.022*	0.162
**BTTS1 vs. BTTS3**	0.354	1.000	0.643
**BTTS1 vs. BTTS4**	0.576	0.571	0.228
**BTTS1 vs. BTTS5**	0.610	0.918	0.640
**BTTS2 vs. BTTS3**	0.065	0.153	0.762
**BTTS2 vs. BTTS4**	0.306	0.194	0.130
**BTTS2 vs. BTTS5**	0.297	0.272	0.371
**BTTS3 vs. BTTS4**	0.867	0.614	0.148
**BTTS3 vs. BTTS5**	0.915	0.933	0.505
**BTTS4 vs. BTTS5**	0.741	0.180	0.171

BTTS = breast tumor tissue selection, ADC = apparent diffusion coefficient (mm^2^/s), AUC = area under the ROC curve, ROC = receiver operating characteristic.

*Significant difference: p <0.05.

### Time measurements

As presented in Table 6, BTTS2 and BTTS3 were the fastest lesion ADC measurement methods. BTTS2 (whole lesion, oval) showed a mean measurement time of 13.4/14.9 seconds (2 observers) and BTTS3 (center 0.3cm^2^, round) 13.8/9.9 seconds (2 observers), compared to mean measurement times of at most 38.8 seconds for BTTS1 (manual whole lesion) by observer 2. The type of BTTS method was of significant influence on the measurement time (p<0.001). Post-hoc pairwise comparison did not show a significant difference between the measurement time of BTTS2 and BTTS3 (p = 1.00), which also applies for BTTS4 and BTTS5 (p = 0.544). The other BTTS methods significantly differed in measurement times (p<0.01).

**Table 6:** Mean measurement time per BTTS methods, observer 1 and observer 2 separately.

	Time observer 1 (sec ±SD)	Time observer 2 (sec ±SD)
**BTTS1**	19.2 ± 5.6 sec	38.8 ± 18.1 sec
**BTTS2**	13.4 ± 4.0 sec	14.9 ± 5.4 sec
**BTTS3**^**a**^	13.8 ± 5.6 sec	9.6 ± 2.1 sec
**BTTS4**^**a**^	25.6 ± 5.9 sec	31.5 ± 15.9 sec
**BTTS5**^**a**^	23.6 ± 5.6 sec	29.0 ± 9.0 sec

BTTS = breast tumor tissue selection. Time is in seconds (sec). SD = standard deviation

^a^ = fixed size.

## Discussion

In this study on the reproducibility, accuracy and measurement time of the most widely used conventional BTTS methods and fixed size tumor delineation, ADC could discriminate benign from malignant lesions. ADCmean showed better overall performance than ADCmin, with good to excellent inter-observer agreement. In the AUC comparison, this study confirms the literature based hypothesis of no significant influence of the BTTS method on the discrimination between benign and malignant breast lesions.

Not a single BTTS method outperformed in lesion differentiation by ADC measurement, due to the high heterogeneity in available data in a recent meta-analysis [14]. The need for robust analysis of BTTS methods in an independent database was evident, especially because of the importance of the breast DWI protocol and image analysis standardization written in the latest consensus statement of the EUSOBI, reporting no consensus on the breast tumor tissue selection method [8].

A comparable high reproducibility of ADCmean for the fixed-size methods (BTTS3-5) with inter- and intra-observer ICCs of 0.882–0.939 is shown in the present study compared to the whole lesion methods (BTTS 1–2), with inter- and intra-observer ICCs of 0.899–0.940. The ADCmean showed higher agreement and AUC than ADCmin measurements with inter-observer ICCs of 0.882–0.940 vs. 0.742–0.875, respectively.

For ADC mean, all 5 BTTS methods showed comparable AUCs, except for BTTS1 vs. BTTS2 for observer 1 reading session 2. The concern that BTTS 1–3 might include the necrotic part of a lesion, which potentially causes false negative results based on higher mean ADC values can be neglected since the BTTS2 (oval shaped, whole lesion) and BTTS3 (standardized fixed volume of 0.12 cm^3^) showed comparable high AUCs of 0.89–0.91 and 0.86–0.88, respectively.

Furthermore, measurement times were shorter for the central volume (0.12 cm^3^) measurement, BTTS3 (13.8/9.6 sec, 2 observers) and the round/oval whole breast tumor tissue selection method, BTTS2 (13.4/14.9 sec, 2 observers) than for the other methods. Therefore, no time consuming methods of conventional manual tumor tissue delineation such as BTTS1 (19.2/38.8 sec, 2 observers) are necessary. Moreover, there is no need to spend time selecting the breast tumor area of lowest diffusion (BTTS 4 and BTTS5) as an indicator of the most cellular part. So far, only Bickel et al. included time measurements as a measure of user’s convenience [9].

This study was performed in accordance with the standardized protocol recommended in the consensus and mission statement of the EUSOBI International breast DWI working group [8]. This protocol consists of axial SS- EPI-DWI with SPAIR fatsupression, a slice thickness of 4 mm, b-values of 0–1000 s-mm^2^ a TR of 9300 ms (>3000), and the lowest possible TE of 91 ms. Bickel et al. showed comparable ICCs for ADCmean and ADCmin for their small and large breast tumor tissue selection methods, with highest ICC for ADCmean with a large tumor tissue selection (inter-observer ICC of 0.85 and intra-observer ICC of 0.89) in comparison to the available literature [9]. Time measurements, with shortest measurement time for a small BTTS methods of 7s (range: 3.3–23.7s) were comparable to those of for BTTS3 (9.6/13.8s) central fixed size measurement in the current study (2 observers). However, they presented higher AUCs for ADCmin (0.95/0.96) than in this study (0.66–0.81). Giannotti et al. showed comparable good inter and intra-observer agreement (0.864–0.997) for ADCmean, with fair inter-observer ICCs of 0.677 for ADCmin in 52 malignant lesions [17]. In the measurement of diffusion, fat containing voxels may show an ultralow ADC value, which could lead to false positive results in benign lesions when using ADCmin as measurement method instead of ADCmean. This partly explains the lower AUC for ADCmin compared to ADCmean, which is illustrated in Table 1 column 5–7, showing relatively low ADCmin values for benign lesions.

Nogueira et al. compared the ADCmean values of 2 observers: inter-observer agreement was excellent for a manual whole lesion selection (ICC = 0.97) and a 10mm^2^ lowest diffusion breast tumor tissue selection (ICC = 0.98), which is higher than in the current study, but measured in significantly fewer (n = 39) lesions [18]. Arponen et al. found a lower intra- and inter-observer agreement: ICC of 0.817 and 0.831 for whole lesion BTTS, respectively, versus 0.707 and 0.589 for lowest diffusion BTTS, respectively [11].

One of the known limitations of DWI is its low spatial resolution. Small lesions, such as small cancer foci, or scattered foci may not be identifiable on DWI. Most studies use a lesion diameter of 1.0 cm as a threshold. Smaller lesions are excluded [19]. No data are available on the minimum size of the lesion that can be detected by DWI, which is dependent on the scanning protocol (slice thickness and interslice gap). By confining our study to lesions larger or equal to 1.0 cm (0.8 cm^2^) and excluding non-mass enhanced lesions, we have limited the influence of partial volume effects on the reported ADC values [15].

Furthermore, it was noted that in some cases the DWI series and the DCE-T1 were visually not correctly linked. This is well known and is due to the difference in slice thickness of DWI and DCE-T1 in particular. To correct for this registration mismatch, BTTS1 was positioned to the right location on the same slice based on anatomical and lesion landmarks. This might have resulted in a slightly lower inter- and intra-observer agreement.

Moreover, the relatively small number of benign lesions (n = 18) compared to the 98 malignant lesions might have caused selection bias. To our knowledge, the use of 1.5T instead of 3.0T is not considered a limitation, because of the proven equal diagnostic accuracy [8, 20].

In this study, the presented breast tumor tissue selection methods all showed fair AUC’s. However, the importance of adding DWI to the breast MRI protocol is to prevent unnecessary biopsies. It is no option yet to replace histological biopsies in the diagnostic algorithm of breast masses with MRI (including DWI) and with MRI as a single diagnostic tool, since for now cancers will be missed. This is a general limitation of DWI in enhancing breast lesions. Histological diagnosis is still required in clinic and remains the gold standard. IVIM or machine learning techniques could be of interest in this matter, and should be addressed more in future studies, for example with the introduction of automated breast tumor tissue selection.

## Conclusion

The performance of fixed-size BTTS methods as a potential tool for clinical decision making shows equal AUC but shorter ADC measurement time compared to manual or oval whole lesion measurements. A fixed size BTTS method is advantageous because of its excellent reproducibility. A central fixed breast tumor tissue volume of 0.12 cm^3^ is the most feasible method for use in clinical practice.
